# Surveillance of Leprosy in Kiribati, 1935–2017 

**DOI:** 10.3201/eid2605.181746

**Published:** 2020-05

**Authors:** Stephen T. Chambers, Nabura Ioteba, Eretii Timeon, Erei Rimon, Helen Murdoch, Jared Green, Emma Trowbridge, Jane Buckingham, Arturo Cunanan, Jonathan Williman, Patricia Priest

**Affiliations:** Christchurch Hospital, Christchurch, New Zealand (S.T. Chambers);; Pacific Leprosy Foundation, Christchurch (S.T. Chambers, N. Ioteba, P. Priest);; University of Otago, Christchurch (S.T. Chambers, J. Williman);; Canterbury District Health Board, Canterbury, New Zealand (N. Ioteba);; Ministry of Health and Medical Services, Bikenibau, Kiribati (E. Timeon, E. Rimon, H. Murdoch);; University of Canterbury, Christchurch (J. Buckingham);; Culion Sanatorium and General Hospital, Culion, Philippines (A. Cunanan);; University of Otago, Dunedin, New Zealand (P. Priest)

**Keywords:** leprosy, crowding, tuberculosis and other mycobacteria, *Mycobacterium leprae*, Kiribati, Makogai, bacteria

## Abstract

In Kiribati, unlike most countries, high and increasing numbers of cases of leprosy have been reported despite the availability of multidrug therapy and efforts to improve case finding and management. Historic records show that 28 cases had been identified by 1925. A systematic population survey in 1997 identified 135 new cases; the mean incidence rate for 1993–1997 was 7.4/10,000 population. After administering mass chemoprophylaxis, the country reached the elimination threshold (prevalence <1/10,000), but case numbers have rebounded. The mean annualized rate of new cases in 2013–2017 was 15/10,000 population, with the highest new case rates (>20/10,000 population) in the main population centers of South Tarawa and Betio. Spread is expected to continue in areas where crowding and poor socioeconomic conditions persist and may accelerate as sea levels rise from climate change. New initiatives to improve social conditions are needed, and efforts such as postexposure chemoprophylaxis should be implemented to prevent spread.

Leprosy (also called Hansen’s disease) was well established in Kiribati by the early 20th century, possibly as a result of contact with Western and Chinese traders (*1*,*2*). Colonial records indicate that there were 28 known cases in 1925, when the population was ≈31,000. Kiribati, formerly the Gilbert Islands, is a country of 33 atolls, 21 of which are inhabited, spread over >1 million square miles of ocean. The country covers an area on both sides of the International Date Line and north and south of the equator. The islands became a British colony in 1916, were occupied by Japan during 1942–1943, and became an independent country in 1979. The population in the 2015 census was 110,136, with the main population located in South Tarawa (39,058 persons) and Betio (17,330 persons) (*3*). Betio, an islet with a deepwater port, is connected to South Tarawa by a causeway.

Leprosy, caused by the bacterium *Mycobacterium leprae*, is a chronic disease with an indolent onset, resulting in a long period between the manifestation of the disease and the person seeking healthcare (*4*). Its 2 clinical forms, paucibacillary disease (PB) and multibacillary disease (MB), may have long-term consequences if untreated and can result in peripheral nerve damage, chronic ulceration, blindness, and facial disfigurement, as well as social isolation and family discord. Complications are more common in MB leprosy (*5*,*6*).

Humans are the main reservoir of *M. leprae.* The primary mode of transmission is understood to be person to person by the respiratory route, but this route has not been proven conclusively (*7*). Patients with MB disease excrete *M. leprae* from their nasal mucosa and skin. Persons most at risk are close household contacts of those with MB, but social contacts are also at risk. Social and economic factors play a role in transmission (*8*). Poverty, undernutrition, crowding, and rapid uncontrolled internal migration have been associated with high rates of leprosy (*9*). Higher rates of leprosy were found in households of >7 persons than those with <4 persons and in homes in which >2 shared a bedroom (*10*,*11*). 

The First International Leprosy Conference, held in Berlin in 1897, adopted segregation as the global response to the threat of leprosy; it was commonly used by colonial governments (*12*). Newly diagnosed patients with leprosy were initially isolated in Kiribati before they were transported to the leprosy isolation island, Makogai, in the Fijian archipelago. Patients from Kiribati were first admitted to Makogai in 1937, although the isolation facility began accepting patients by 1911. Dapsone, the first effective drug to treat leprosy, became available in 1945 and enabled patients on Makogai to be treated and repatriated. The leprosy isolation facility was closed in 1969. Multidrug therapy (MDT) consisting of rifampin, dapsone, and clofazamine was successfully introduced to Kiribati by 1990 (*13*).

Optimism about the efficacy of MDT and evidence of leprosy control led the 44th World Health Assembly to adopt resolution WHA44.9 in May 1991 to eliminate leprosy as a public health problem by the year 2000 (*14*). The elimination target was defined as a prevalence of <1 case/10,000 population. For calculating prevalence, cases were defined as patients registered for MDT treatment, which reflected the burden of disease. Concern that Kiribati would not meet the elimination goal was raised in 1996 when leprosy was found to be highly endemic to Kiribati, and mass screening of the whole population was conducted in 1997 and repeated in 1998, supported by the World Health Organization (*15*). Chemoprophylaxis (single-dose rifampicin, ofloxacin, and minocycline) was administered to the population of South Tarawa, including Betio, and Christmas Island (*15*). Reported cases fell following this intervention, and Kiribati reached the elimination goal in 2000 (prevalence 0.94 cases/10,000 population). However, prevalence has since rebounded above elimination levels, with high numbers of new cases among children, a marker of transmission (*16*). Leprosy has also been identified among Kiribati nationals who have moved to neighboring countries including the Solomon Islands, Fiji, and New Zealand (*4*). Leprosy control is recognized as a priority by the Government of Kiribati (*17*). We describe the rates of new cases of leprosy from historic and recent medical records to document emergence and transmission of leprosy in Kiribati. 

## Methods

We aimed to use the most reliable surveillance data sources available for this study. One source was the records of patients admitted to Makogai isolation island in Fiji. Patients with presumed leprosy were identified in Kiribati and sent to Makogai, where a leprologist examined them and validated the diagnosis. Those with leprosy were then interned on Makogai. After the isolation facility was closed, the case records of all patients were transported to the Patrick Twomey Memorial Leprosy Hospital in Suva, Fiji, where they were stored and later entered into an electronic database. 

A second source of information was the medical records of the National Leprosy Unit of Kiribati, which is located in the only secondary medical facility in Kiribati. All cases of leprosy in Kiribati are referred to this center, which is responsible for validating cases, reviewing complex cases, and ensuring medication is sent to patients across the country and which maintains clinical records.

### Population Statistics

We obtained population statistics on numbers and crowding from census data available online for 2005, 2010, and 2015, and in hard copy for 1990 and 2000, from the national statistics office under the Ministry of Finance of the Government of Kiribati and the World Bank (*3*,*18*). We obtained national income from the World Bank figures in current US dollars.

### Case Definition

A case of leprosy is defined as illness in a person who has >1 features and who has not completed a full course of treatment. The features are the following: hypopigmented or reddish lesions with definite loss of skin sensation; involvement of peripheral nerves, as demonstrated by definite thickening with definite loss of skin sensation; and detection of acid-fast bacilli in the skin by biopsy or slit skin smear (*19*). Cases were classified into PB disease and MB disease in accordance with WHO criteria. Grade 2 disability was defined as visible deformity or damage present in the hands or feet or severe visual impairment (vision worse than 6/60; inability to count fingers at 6 m; lagophthalmos, iridocyclitis, or corneal opacities) (*19*,*20*). 

### Record Sources

We (J.B.) compiled, checked, and locked an electronic database from the medical records of case-patients from Kiribati admitted to Makogai (1935–1964). The records have been held at the Twomey Leprosy Hospital in Suva, Fiji, by the Pacific Leprosy Foundation (PLF), a nonprofit organization that supports leprosy prevention, treatment, and patient welfare work around the Pacific, working in partnership with the Ministries of Health under a memorandum of understanding.

We obtained the case registers from the National Leprosy Unit (NLU) at the Nawerewere hospital in Kiribati and entered information on cases from 1988–2010 into a separate database. We (S.T.C.) checked the accuracy of data entry (90%). In 2010, staff at the NLU began entering data prospectively. We backed up this database to cloud-based storage weekly, and the PLF checked for completeness, double entries, and other errors. In addition, PLF staff review the data on regular site visits. These records are the source documents for WHO reports. 

To diagnose leprosy, the medical and nursing staff of the NLU perform clinical examination of patients in North and South Tarawa in person, and by radio consultation with medical assistants and nurses on the outer islands. In 1997–1998, all cases were seen and validated by leprologists involved in a nationwide screening program that covered 92.2% of the population (*15*). In 2007–2017, a consultant leprologist supported by the PLF validated cases during regular visits.

### Intensification of Case Finding

Because of concern that the number of new cases was increasing, we intensified the control program beginning in 2008. A consultant leprologist (A.C.) visited Kiribati every 3–4 months and conducted regular educational workshops for medical assistants, nurses, and staff in the NLU.

We introduced active case finding for 1 year in 2010, followed by ongoing publicity campaigns that included visits to village meeting houses by drama groups, radio advertising, and a song recorded by a local musician. Since 2015, health promotion activities have been intensified; we held free dermatology clinics in areas of apparent high leprosy case load in South Tarawa and Betio. Active screening of household contacts of leprosy patients began in 2016. 

### Statistical Methods

We performed statistical analyses using Microsoft Excel (Microsoft, https://www.microsoft.com) and R statistical software (*21*). We summarized cases by year, age, and clinical form. We calculated crude rates with 95% Wilson binomial CIs by dividing counts by the population estimates obtained from the World Bank. We standardized rates by age, using 5-year categories, to the WHO’s world 2000–2025 population. We estimated incidence rate ratios comparing age and sex, stratified by clinical form and adjusting for year, using Poisson regression models. 

### Ethics Considerations

The Ministry of Health and Medical Services in Kiribati approved the study design. The ethics committee of the University of Otago, Christchurch, New Zealand, approved the study. 

## Results

### Population of Kiribati

Kiribati has been experiencing rapid population growth and increasing concentration of people in South Tarawa and Betio, where the percentage of the population rose from 5% in 1947 to 51% in 2015 (Table 1) (*3*,*18*). We observed a corresponding increase in population density, but numbers per household were relatively stable over time (Table 2). The number of occupants per household was higher in South Tarawa and Betio than the outer islands of Kiribati. GDP per capita increased 300% between 1990 and 2015, but from a very low base (from US $550 to US $1,648; current dollars, World Bank data) (*18*).

**Table 1:** Population of Kiribati and the population centers of South Tarawa and Betio, 1931–2015

Census year	Kiribati population	South Tarawa and Betio population (% total population)
1931	29,751	3,013 (10)
1947	31,513	1,617 (5)
1963	43,336	6,109 (14)
1968	47,735	10,616 (22)
1973	51,926	14,861 (29)
1978	56,213	17,921 (32)
1985	63,883	21,393 (33)
1990	72,335	25,380 (35)
1995	77,658	28,350 (37)
2000	84,494	36,717 (43)
2005	92,533	40,331 (44)
2010	103,058	50,182 (49)
2015	110,110	56,324 (51)

**Table 2:** Demographic and leprosy data in Kiribati by region and census year*

Location	Years	Population at census date	Population density, persons/km^2^	No. persons in household	No. cases	No. cases/10,000 persons/y
South Tarawa	1988–1992	15,937	1,106	7.6†	58	7.3
	1993–1997	18,006	1,250	8.0†	86	9.6
	1998–2002	24,449	1,697	8.1†	42	3.4
	2003–2006‡	27,802	1,891	7.7†	64	5.6
	2009–2012‡	34,427	2,443	7.3	215	15.6
	2013–2018	39,058	2,772	7.0	403	20.6
Betio	1988–1992	9,443	5,555	NA	36	7.6
	1993–1997	10,344	6,085	NA	56	10.8
	1998–2002	12,268	7,216	NA	35	5.7
	2003–2006‡	12,509	7,358	NA	39	7.8
	2009–2012‡	15,755	9,434	8.0	94	14.9
	2013–2018	17,330	10,377	7.6	234	27
Outer Islands	1988–1992	46,161	65	6.4	153	6.6
	1993–1997	49,308	69	5.9	144	5.8
	1998–2002	47,777	67	5.7	48	2
	2003–2006‡	52,222	73.5	5.9	57	2.7
	2009–2012‡	52,876	74	5.7	189	8.9
	2013–2018	53,748	76	5.4	199	7.4
Total for Kiribati	1988–1992	72,335	88.3	7.8	250	6.9
	1993–1997	77,658	95.8	7.23	286	7.4
	1998–2002	84,494	104.2	7.4	125	3
	2003–2007	92,533	114.1	6.6	224	4.8
	2008–2012	103,058	127.1	6.4	566	11
	2013–2018	110,136	135.8	6.2	836	15.2

*Population data shown are taken from the national Kiribati census, which reports every 5 years starting from 1990. Leprosy case data are taken from the register at the National Leprosy Unit of Kiribati and the average number of cases for a 5-year period using the census year as the central value. NA, not available.  †Includes data for Betio from 1990–2005. ‡For the years 2007 and 2008, the location data were incomplete. Rates have been reported for 4 years with complete data. Data for all of Kiribati for 2007 and 2008 were available.

### Cases from Makogai Case Register

The database recorded 236 patients admitted to Makogai with leprosy. Of these, 87 were admitted during 1935–1940; another 5 during 1942–1946, during and immediately after World War II; and 141 during 1947–1954. The last 4 patients were admitted during 1956–1964, before admissions were stopped. Of the 236 cases, 67 were classified as cutaneous/tuberculoid, 121 as lepromatous, 47 as neural leprosy, and 1 as indeterminate. The Kiribati population was relatively stable from 1931–1947 at ≈30,000 persons, giving an annual new case rate of 3.9/10,000 population.

### Cases from the National Leprosy Unit Case Register, 1988−2017

No case records were archived before 1988. During 1988–2017, a total of 2,287 new cases were reported in Kiribati, 1,242 (54%) of which were in male patients. A total of 757 cases (33%) were MB, and 750 (33%) were in patients <15 years of age. Of the MB case-patients, 63% were male. Grade 2 disability was reported in 46 (3%) of the 1,338 cases reported from 2009–2017; the data are inconsistent before 2009.

**Figure 1:**
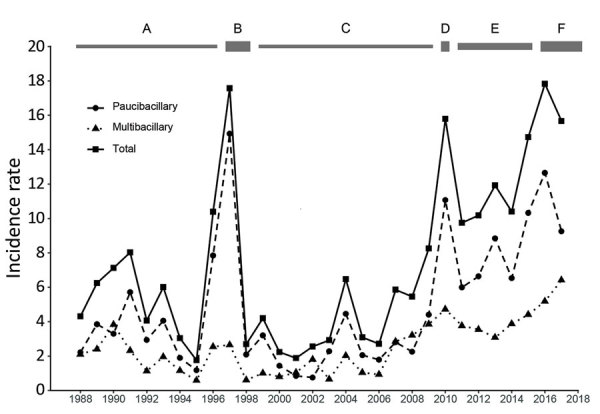
Age-standardized incidence rates (cases/10,000 population) of leprosy recorded, by year and type, from the case register of the National Leprosy Unit, Nawerewere Hospital, Kiribati, 1988–2018. Bars at top indicate timing of passive case finding (A and C), a national screening program (B), active case finding (D), an intensified awareness program (E), and case finding in household contacts (F).

The large number of new cases reported in 1997 was because of the nationwide screening and treatment campaign (92.2% of the population), precipitated by the rise in cases seen in 1996 and the adoption of the WHO elimination target of a prevalence of <1/10,000 population by 2000 (Figure 1). South Tarawa and Betio were screened again in 1998, covering 90.3% of the population. These efforts identified a large number of cases in South Tarawa and Betio rather than in the Outer Islands, which had previously been the site of most cases. The spike in reported cases in 2010 was from active case finding over that year. During 2009–2017, Betio and South Tarawa together contributed 786 (76%) of reported cases. The overall rate of both PB and MB leprosy rose with time, and the percentage of PB diagnosed increased in times of active surveillance and fell when surveillance was stopped.

**Figure 2:**
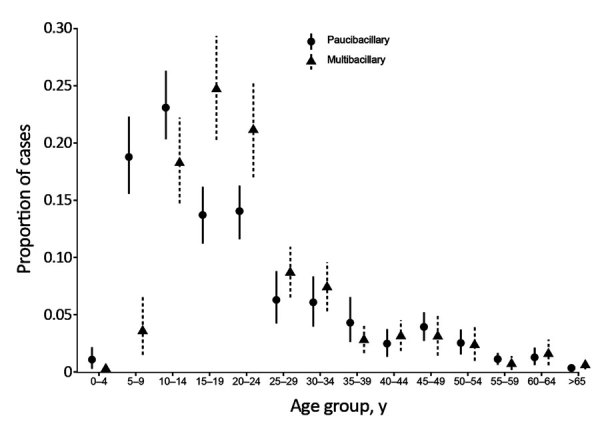
Distribution of cases of leprosy, by age group and type, Kiribati, 1988–2018. Points represent the pooled mean proportion of cases by age. Vertical lines represent bootstrapped 95% CIs.

**Figure 3:**
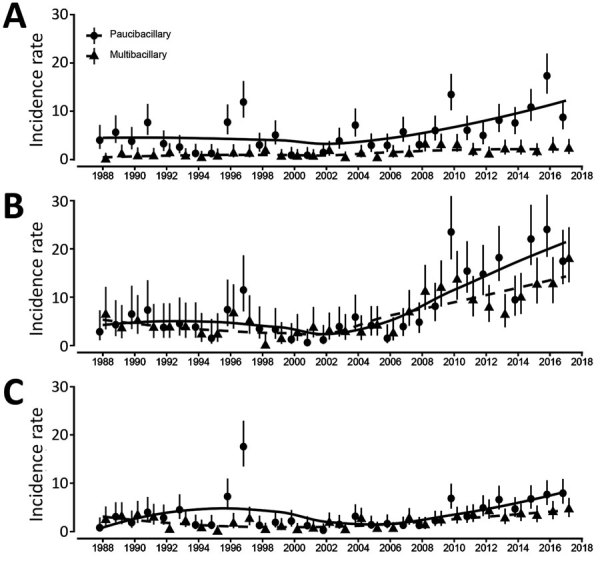
Age-specific incidence rates (cases/10,000 population) for multibacillary and paucibacillary leprosy, by age group, Kiribati, 1988–2018. A) Age 0–14 years; B) 15–24 years; C) 25–64 years. Points represent the age-specific rate and vertical lines 95% CIs. Solid lines indicate the locally estimated scatterplot smoothing moving average of age-specific incidence rates of paucibacillary leprosy; dashed lines, of multibacillary leprosy.

The age distribution of MB and PB patients has remained relatively stable over time. We pooled the data to demonstrate the mean percentage of cases by age at diagnosis for MB and PB. Our findings showed that PB was diagnosed more frequently than MB in children <10 years of age and MB more frequently in patients 15–24 years of age (Figure 2). Changes in age-specific rates over time demonstrate that the rates of leprosy have been increasing in all age groups (Figure 3). Estimates from Poisson regression models suggested that incidence rates of MB in those 15–65 years of age were twice as high in male patients as in female patients (incidence rate ratio 2.1, 95% CI 1.7–2.4; p<0.001). We saw no difference in rates by sex for those with MB disease <15 years of age (incidence rate ratio 1.1, 95% CI 0.79–1.5; p = 0.59) and no difference in incidence rate ratio by sex for PB disease (p = 0.76).

The mean annual number of cases for a 5-year period for Kiribati has risen from 6.9/10,000 population in 1988–1993 to 15.2/10,000 population in 2013–2017 (Table 2). We grouped new diagnoses by location over 5-year periods using the census date as the center point; our analysis was limited because location was inconsistently recorded for the years 2007 and 2008, although age, sex, and classification data were complete. We omitted these 2 years from the location analysis (Table 2). The new case rate increased from 7.6/10,000 population/year in 1988–1992 to 27.0/10,000 population/year in 2013–2018 in Betio, from 7.3/10,000 population/year to 20.6/10,000 population/year in South Tarawa, and from 6.6/10,000 population/year to 7.4/10,000 population/year in the Outer Islands. The increase in case rate occurred at a similar time as the increase in overall population density, whereas the number of persons per household was stable.

## Discussion

Control of leprosy in Kiribati has never been achieved. Initial control efforts by isolation of known leprosy cases in Kiribati were clearly documented in the medical records from Makogai; these records indicate a new case rate of ≈4/10,000 population/year in Kirabati. The incidence rate is almost certainly an underestimate given the stigma associated with leprosy and the tendency to avoid persons with leprosy, transport them away from their families, and isolate them on an island thousands of miles away from home. Despite the introduction of dapsone, closure of Makogai, and widespread use of MDT, the elimination of leprosy as a public health problem was only reached in 1999 and has not been maintained in Kiribati. Rather, the number of cases has continued to rise since 1999. The speed and scale of the increase in cases demonstrate the potential for case numbers to rebound.

Unsurprisingly, increased case finding efforts have identified more cases in Kiribati. Surveys in India and Brazil have similar findings, which has raised concerns that cases may remain undetected even in areas of apparently low endemicity (*22**–**24*). The intensive activity in 1997 of Daulako et al. was a landmark event; they screened >90% of the population of Kiribati (*15*). Although the number of new cases dropped dramatically after this intervention, temporarily reaching the elimination target, case numbers have steadily climbed since. A combination of factors, such as a belief that leprosy had reached the WHO elimination target and was therefore defeated, a temporary effect of single dose prophylaxis administered in 1998, lack of active case finding, and low-resource status, may have contributed to this change. 

The increase in annualized rate of new cases beginning around 2009 is most notable in South Tarawa and Betio, but a rise was also reported from the outer islands. The number of cases reported in the outer islands may be an underestimate, given that the increased detection, treatment and control efforts have been focused on South Tarawa and Betio. The data suggest that the conditions for spread persist in the outer islands but may be worsening in South Tarawa and Betio because of internal migration and worsening of socioeconomic conditions (*8**–**10*).

The percentage of cases of PB disease and low reported percentage of disability are consistent throughout 1988–2017. The period includes the well-documented whole-population survey in which Daulako observed similar findings (*15*). The high percentage of PB disease and low percentage of disability found in Kiribati are consistent with reports from other Micronesia countries, such as the Marshall Islands and the Federated States of Micronesia, which share similar demographic and socioeconomic characteristics (*16*).

In Kiribati, 32% of all new case-patients during 1988–2017 were children. One of the highest reported worldwide, this rate indicates a failure to control transmission. Other reports of high national percentages among children, including those from the Marshall Islands (15/80, 19%), the Federated States of Micronesia (40/169, 24%), Papua New Guinea (89/356, 25%), and Solomon Islands (7/43, 16%), indicate that conditions for transmission are not limited to Kiribati but are widespread in other regions of the Western Pacific region (*16*). 

The high rate of leprosy in Kiribati is probably related to the socioeconomic conditions, but these relationships are not well understood. Household crowding has been associated with high rates of leprosy in both Brazil and Indonesia (*10*,*11*). Households of more than 7 persons, which is common in Kiribati and particularly in Betio and South Tarawa, appear to be at risk for leprosy infection. High rates of disease are also reported in isolated populations and those marked by displacement and civil unrest that may increase crowding and poverty, both of which are associated with transmission of *M. leprae* (*25*,*26*). The marked increases in the populations in the urban and semiurban areas of both South Tarawa and Betio have been driven by increased opportunities for work in Betio, with development of the port and light industry, and in South Tarawa, the location of central government and the international airport. Crowding caused by limited land availability and single-story homes has amplified the risk for spread of leprosy as well as other infections, such as tuberculosis, which is also reported at a high rate in Kiribati (*27*). These conditions may exacerbate deficiencies in healthcare services such that clinical infection remains unrecognized and untreated for prolonged periods. 

Poor nutrition plays a role in susceptibility to leprosy. Case control studies in Brazil, India, and Bangladesh have identified food insecurity, intermittent starvation, and a lack of diversity in the diet as contributors to a high rate of leprosy (*28**–**30*). Pediatric undernutrition, maternal obesity, and micronutrient deficiencies are present in Kiribati. Children <5 years of age are particularly at risk; 34% are reported to have stunted growth and 37% to have anemia (*31**–**32*). A recent study demonstrated that low dietary diversity and a high prevalence of multiple micronutrient deficiencies were common in Kiribati (*33*). 

Economic conditions in Kiribati, although improving, are rising from a low base; we expect to see substantial pressures on economic resources and land use associated with climate change and sea level rise, as well as population increase. These changes may have substantial effects on living standards and leprosy rates. 

The limitations of our analysis include the potential for error in diagnosis, case recording, and data transcription. To mitigate the risk for errors, we have validated cases by a leprologist, recorded data prospectively, and checked the entered data against the case registers. Underdiagnosis of cases is likely but will have been reduced with active case finding to identify previously unsuspected cases. Overall, the changes in rates suggest that our observations are sufficiently robust to indicate real changes in the spread of leprosy in Kiribati.

In conclusion, the number of new cases and age-standardized rates of leprosy reported in Kiribati have risen over the past decade, despite the ready availability of MDT. The long incubation period for leprosy implies that it may reemerge and rates increase if conditions such as crowding worsen, if economic development is not achieved, or if leprosy services are poorly resourced. Reaching the WHO-specified elimination goal may be temporary without an ongoing commitment to comprehensive control programs over the long term (*34**–**36*). The introduction of postexposure prophylaxis to household contacts or to high-risk populations may offer a new tool to reduce the number of cases, the social consequences of stigma, and disability; this treatment has begun in several poorly resourced countries (*37**–**40*). 
